# Severe Short Stature and rhGH Resistance in a Child Born SGA: The Role of a Novel IGF1R Mutation, Case Report and Narrative Review

**DOI:** 10.3390/children13040458

**Published:** 2026-03-27

**Authors:** Giovanni Luppino, Eleonora Ini’, Letteria Anna Morabito, Tiziana Abbate, Cecilia Lugarà, Tommaso Aversa, Malgorzata Wasniewska, Domenico Corica

**Affiliations:** 1 Department of Human Pathology of Adulthood and Childhood, University of Messina, Via Consolare Valeria 1, 98125 Messina, Italy; eleonoraini22@gmail.com (E.I.); letteria.morabito@gmail.com (L.A.M.); tiziana.abbate93@gmail.com (T.A.); cecilialug@gmail.com (C.L.); tommaso.aversa@unime.it (T.A.); malgorzata.wasniewska@unime.it (M.W.); 2 Pediatric Unit, AOU Policlinico G. Martino, Via Consolare Valeria 1, 98125 Messina, Italy

**Keywords:** growth hormone therapy, IGF1 serum levels, IGF1R gene mutation, p.Tyr1166Ter variant, small for gestational age

## Abstract

**Highlights:**

**What are the main findings?**

- Blunted growth response and lack of catch-up after 3 years of rhGH in a child born SGA and with a novel variant of the *IGF1R* gene (c.3498C>G; p.Tyr1166Ter).
- The onset of glucose alterations during rhGH therapy highlights the possible interplay between the patient’s SGA background, *IGF1R* mutation, and GH-driven metabolic stress.

**What are the implications of the main findings?**

- Growth outcomes in the IGF1R gene defects are significantly lower than those of SGA children with short stature without identifiable genetic defects.
- The convergence of SGA status and intrinsic IGF1R resistance creates a synergistic metabolic risk, which could be further exacerbated by rhGH therapy acting as a metabolic stressor.

**Abstract:**

**Background**: Genetic causes of growth failure should be suspected in patients born small for gestational age (SGA) who fail to show postnatal catch-up growth, present with severe short stature (SS), and exhibit a poor or absent response to growth hormone (rhGH) therapy. Mutations in the insulin-like growth factor 1 receptor (IGF1R) gene are associated with impaired growth, intrauterine growth restriction (IUGR), low birth weight and/or length, and postnatal SS. Case Description: A 9-year-old boy, born SGA for birth length, was evaluated for severe SS. Common causes of SS were excluded. At 9 years and 7 months of age, his height was 112.6 cm (−3.99 SDS), weight 18 kg (−3.79 SDS), and BMI 14.2 kg/m^2^ (−1.8 SDS); pubertal development was Tanner stage 1. The target height was 158 cm (−2.62 SDS). Bone age was delayed by approximately one year compared with chronological age. Serum IGF-1 levels were within the upper-normal range for age. GH therapy (0.035 mg/kg/day) was initiated due to the lack of catch-up growth in an SGA subject. After three years of treatment, the height gain was only 0.5 SDS. IGF-1 levels showed a transient treatment-related increase, followed by persistent normalization during ongoing therapy. Next-generation sequencing (NGS) analysis identified novel heterozygous paternal nonsense variant in the *IGF1R* gene: c.3498C>G (p.Tyr1166Ter). At 12 years of age, impaired fasting glucose and reduced glucose tolerance were detected; consequently, it was decided to discontinue rhGH therapy, also in light of the *IGF1R* mutation and the lack of height recovery. **Conclusions**: This case underlines the critical role of genetic testing in the evaluation of patients born SGA. The coexistence of SGA status and an *IGF1R* gene mutation may provide a clear explanation for both the poor response to rhGH therapy and the increased risk of alterations in glucose metabolism. An extensive narrative review of the literature on growth outcomes and glucose metabolism abnormalities during GH treatment in SGA patients carrying *IGF1R* variants was also performed.

## 1. Introduction

Small for gestational age (SGA) is defined as a birth weight and/or length below −2.0 standard deviation scores (SDS) for gestational age. SGA comprises a heterogeneous group, including children with various conditions resulting from a complex combination of maternal, placental, fetal, and genetic factors, which may result in low birth weight and length [1]. During the first two years of life, clinical management focuses on optimizing nutritional intake to promote adequate catch-up growth, while preventing metabolic complications such as excessive weight gain [2]. In addition, although most SGA infants could have compensatory growth by the age of two, about ~10% persist in a condition of persistent short stature (SS), that is defined as a height ≤ −2 SDS below the mean of a specific population adjusted for age and sex [3]. Children with SS and born SGA without postnatal growth recovery may be eligible for recombinant human growth hormone (rhGH) treatment, once other causes of growth delay have been excluded [4].

SS represents a multifactorial condition arising from a broad spectrum of physiological, pathological, endocrinological, nutritional, and genetic determinants [5]. Genetic causes of growth impairment should be suspected in the presence of auxological features, including severe and disproportionate SS, associated dysmorphic traits, neurodevelopmental delay, or congenital organ malformation, and in cases of poor or absent response to growth-promoting therapies [6]. Previously, SS in SGA cases was often labeled as an idiopathic condition, whereas today several monogenic conditions have been recognized in this population [7]. Nowadays, the expanding availability of targeted gene panels, chromosomal microarray analysis, Next Generation Sequencing (NGS), and whole-exome sequencing revealed a broad spectrum of genetic contributors to SS [8]. Monogenic growth disorders are caused by harmful changes in a single gene that disrupt the normal process of growing. Among the genes most frequently implicated are those that affect growth plate development (*SHOX*, *NPR2*, *ACAN*, *FGFR3*, *IHH*), as well as genes that modulate GH secretion, GH receptor function, IGF-1 synthesis and signaling, and GH–IGF-1 axis (*GH1*, *GHR*, *GHRHR*, *STAT5B*, *IGF1*, *IGF1R*, *IGFALS*) [9].

The type I insulin-like growth factor receptor gene, located on 15q26, encodes the type-1 insulin-like growth factor receptor (IGF1R). This ligand-activated tyrosine kinase mediates most of the growth-promoting effects of circulating and locally produced IGF-1, both prenatally and postnatally [10]. Genetic alteration of *IGF1R*, including mutations or deletions, may affect receptor expression, processing, ligand binding, or downstream signaling, leading to partial or complete IGF-1 resistance [10,11]. These genetic mutations ultimately impact the growth plate, resulting in insufficient growth, intrauterine growth restriction (IUGR), low birth weight/length, microcephaly, and persistent postnatal SS [11].

This article presents a rare clinical case of severe SS in a child born SGA for length, carrying a novel pathogenic *IGF1R* variant, and showing resistance to daily rhGH therapy. Furthermore, we provide a narrative review of the scientific literature regarding the available evidence on height growth outcomes, the use of rhGH therapy, and the potential presence of glucose metabolism alterations in SGA children carrying *IGF1R* gene mutations or 15q deletions.

## 2. Case Description

A 9-year-old boy was referred to the pediatric endocrinology outpatient clinic for severe SS and decreased growth velocity. He was born at term (39 weeks of gestation) to non-consanguineous parents, following an uncomplicated pregnancy. Birth weight was 2800 g (−1.66 SDS) and birth length was 45 cm (−2.81 SDS); according to Bertino’s growth charts [12], he was classified as SGA for birth length.

The family history was negative for endocrine and metabolic disorders. Both parents were healthy; their heights were 149.0 cm (mother) and 154.0 cm (father). The calculated target height of the proband was 158 cm (−2.62 SDS).

At initial assessment, the auxological evaluation confirmed severe SS. Phenotypically, the child showed no dysmorphic features and exhibited proportionate body proportions. Height was 112.10 cm (−3.99 SDS), weight 18.2 kg (−3.64 SDS), and BMI 14.48 kg/m^2^ (−1.65 SDS) according to Italian growth charts. The child was prepubertal (Tanner stage 1). Bone age assessed by the Greulich and Pyle method, was 8 years, showing a delay of approximately 1 year compared with chronological age.

A comprehensive diagnostic workup was performed to investigate potential causes of growth failure. Endocrine, hematologic, nutritional, and gastrointestinal conditions were excluded, including thyroid dysfunction and celiac disease. Glucose metabolism parameters (fasting glucose and insulin, glycated hemoglobin) were normal at the initial evaluation. A glucagon stimulation test was performed to evaluate GH secretion, demonstrating a normal peak value (18.34 ng/mL), excluding a GH deficiency in accordance with Italian guidelines [13]. At first evaluation, serum IGF-1 levels were within the upper-normal range of the age-adjusted reference (220 ng/mL; +1.7 SDS according to Horenz 2021) [14].

**Figure 1:**
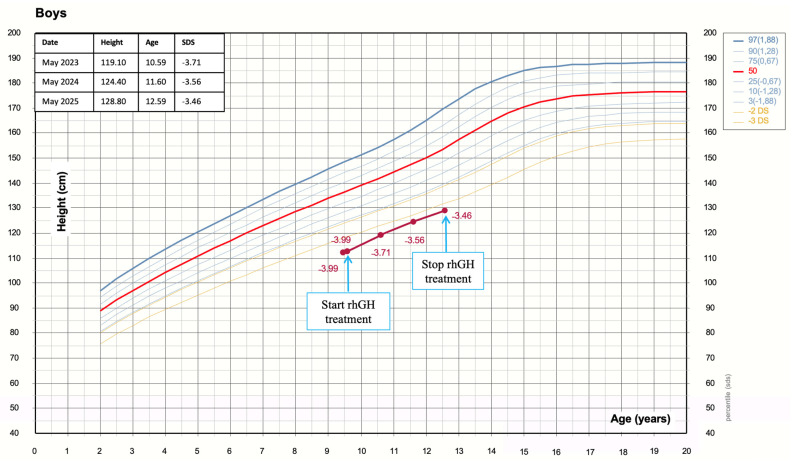
Growth charts of height (SDS) during the follow-up.

Given the severity of growth impairment and the familial pattern of SS, NGS was performed. The targeted gene panel included genes involved in growth regulation and the GH–IGF-1 axis (*GH1*, *GHR*, *GHRHR*, *IGF1*, *IGF1R*, *STAT5B*, *FGFR3*, *IHH*, *ACAN*, *SHOX*, and *NPR2*). Pending genetic results, treatment with rhGH was initiated at a dose of 0.035 mg/kg/day, indicated for SGA without spontaneous catch-up growth, in accordance with Italian guidelines for GH therapy [13]. Treatment was started at 9 years and 7 months of age, at which time his height was 112.6 cm (−3.99 SDS), weight 18 kg (−3.79 SDS), and BMI 14.2 kg/m^2^ (−1.8 SDS). The patient demonstrated excellent treatment compliance and was closely monitored for both auxological and metabolic parameters. (Table 1). Growth rate during treatment is summarized in Figure 1.

After three years of continuous GH treatment, the expected catch-up growth had not occurred, with a total height recovery of only 0.5 SDS. Particularly, at 12 years and 7 months of age, height was 128.8 cm (−3.46 SDS), weight 25.30 kg (−3.27 SDS), and BMI 15.25 kg/m^2^ (−1.93 SDS). Annual growth velocity over the last year was 4.42 cm (−1.12 SDS). The child was prepubescent (Tanner stage G1P1). Bone age indicates a delay of approximately 2 years relative to chronological age. Serum IGF-1 level was 392 ng/mL (+1.3 SDS). Longitudinal evaluation demonstrated that baseline IGF-1 values were within the normal range, albeit close to the upper limit. After initiation of GH therapy, IGF-1 levels exhibited a transient treatment-related elevation, followed by sustained normalization throughout the course of therapy.

The NGS analysis identified a novel heterozygous paternal nonsense variant in the *IGF1R* gene: c.3498C>G (p.Tyr1166Ter). The variant was not previously described in the literature nor reported in the gnomAD and ClinVar population databases. This *IGF1R* gene mutation was classified as likely pathogenic based on in silico predictive models and familial segregation, as both the proband and his father harbor the same mutation and exhibit a consistent phenotype, and according to American College of Medical Genetics and Genomics (ACGM) guidelines, as also reported on the Varsome and Franklin database.

Pathogenic variants in the *IGF1R* gene are known to impair both prenatal and postnatal growth and may predispose to abnormalities in glucose metabolism, reflecting the essential role of IGF-1 receptor signaling in growth and metabolic regulation. In light of this finding, an oral glucose tolerance test (OGTT) was performed during GH treatment, revealing impaired fasting glucose (fasting plasma glucose 102 mg/dL) and impaired glucose tolerance (2 h plasma glucose 145 mg/dL) with normal insulin secretion (fasting plasma insulin 12.5 uIU/mL, 2 h plasma insulin 59.5 uIU/mL), despite the patient following a balanced diet (Mediterranean diet). In light of the poor growth response to GH therapy, the presence of metabolic alterations, and the coexistence of further metabolic risk factors (namely IGF-1 resistance and SGA status), the multidisciplinary team decided to discontinue rhGH treatment at 12 years and 7 months of age.

## 3. Discussion

This case describes the linear growth pattern during a 3-year follow-up of a child born SGA for length and carrier of a novel *IGF1R* gene mutation (p.Tyr1166Ter), who was refractory to GH therapy and developed metabolic complications, potentially linked to the underlying genetic background and possibly exacerbated by rhGH therapy.

Genetic pathways in the determinism of IUGR and SGA conditions play a determinant role [15]. The prevalence of heterozygous *IGF1R* variants or 15q deletions is estimated at 1% to 2% among SGA children with SS [16]. The clinical profile of patients with 15q deletions is complex and characterized by pre- and postnatal growth retardation, cardiac disease, intellectual disability, diaphragmatic hernia, hearing problems, and neonatal lymphedema. Moreover, peri-natal growth retardation, microcephaly, and elevated levels of serum IGF-1 are the main clinical elements in cases with variants of the *IGF1R* gene [17]. In addition, epigenetic and the single-nucleotide polymorphism (SNP) of the *IGF1R* gene can impact the determinism of fetal and neonatal growth failure. From the analysis of 225 SNPs in 10 genes involved in growth and glucose metabolism in 1437 children, including 633 born SGA, the minor allele A of SNP rs4966035 of *IGF1R* was significantly more prevalent among SGAs and is significantly associated with birth length, regardless of gestational age [18]. Thus, a single gene mutation of *IGF1R* should be suspected in short SGA children with microcephaly and altered serum IGF-I concentrations [19].

Regarding the growth rate in patients with *IGF1R* gene mutation, several studies and case reports suggest a less favorable response to treatment with rhGH than SGA patients without genetic mutation.

Göpel et al. analyzed growth and response to rhGH in SGA patients carrying the *IGF1R* mutation and SGA children without a genetic mutation. Compared to SGA controls, *IGF1R* mutation carriers were significantly shorter at rhGH start (median −3.41 vs. −2.71 SDS) and showed a markedly blunted growth response during the first year (+0.87 vs. +3.29 SDS). Moreover, while no significant differences in HOMA-IR were observed at baseline, *IGF1R* mutation carriers exhibited a significantly greater increase in insulin resistance during treatment compared to the SGA cohort [20].

Walenkamp J.E. et al. [21] evaluated the response to rhGH therapy during the first three years of treatment in 12 patients with pathogenic/likely pathogenic *IGF1R* variants (Group 1) and 7 patients with terminal 15q deletions (Group 2), compared to SGA children with SS and no known genetic mutations. The mean height gains in Groups 1 and 2 were 0.9 and 1.3 SDS, respectively, compared to 1.8 SDS in the SGA controls; this suggests that children with *IGF1R* defects show a lower response compared to SGA patients. Furthermore, subjects with genetic mutations exhibited a marked increase in serum IGF-1 levels during rhGH treatment. Notably, those with terminal 15q deletions showed significantly higher IGF-1 concentrations than patients carrying an *IGF1R* mutation. This suggests that a complete gene loss due to deletion leads to a more profound defect than that caused by a single point mutation [21].

Klammt et al. reported consistent findings regarding first-year growth velocity and serum IGF-1 levels in six patients with *IGF1R* mutations [22]. Also, several case reports highlight the growth rate in SGA patients with *IGF1R* gene mutation during the GH treatment, and data are summarized in Supplementary Materials Table S1 [16,23,24,25,26,27,28,29].

These findings are generally consistent with our case, although our patient did not exhibit markedly elevated serum IGF-1 levels. This discrepancy might be explained by the specific nature of the *IGF1R* mutation (p.Tyr1166Ter); being a nonsense mutation, the premature stop codon may trigger nonsense-mediated mRNA decay. This surveillance mechanism degrades defective transcripts to prevent the translation of truncated proteins, likely resulting in a near-total absence of functional receptors on the cell membrane. Such a profound receptor deficit could explain the lack of the expected compensatory IGF-1 elevation typically observed in less severe receptor defects [30]. Based on the genomic position of the p.Tyr1166Ter variant (exon 19), the activation of the nonsense-mediated mRNA decay (NMD) mechanism is highly probable, consistent with literature reports for *IGF1R* nonsense mutations located upstream of the final exon [31].

Furthermore, our patient developed alterations in glucose metabolism during rhGH therapy. This clinical finding is particularly noteworthy as the patient was born SGA, a condition that independently carries an increased risk of impaired glucose tolerance (IGT) and insulin resistance due to metabolic programming [32]. Moreover, the intrinsic *IGF1R* defect leads to an impairment of the shared signaling pathways between IGF-1 and insulin, often predisposing individuals to insulin resistance. Our hypothesis of a link between rhGH therapy and the coexistence of SGA and *IGF1R* mutation in the onset of glucose alterations is supported by existing scientific data.

Burkhardt et al. reported glucose metabolism alterations in two brothers born SGA and their father, all harboring a novel heterozygous *IGF1R* mutation (p.Cys1248Tyr). The eldest brother developed impaired glucose tolerance (IGT) after two years of rhGH therapy, whereas the younger sibling presented with IGT before treatment initiation. Their father was diagnosed with type 2 diabetes mellitus in adulthood [25]. A variable metabolic phenotype ranging from normal glucose tolerance to IGT and fasting hyperglycemia in association with insulin resistance was detected in four Italian family members carrying a novel *IGF1R* mutation (p.Tyr387X) [10]. In this context, the administration of rhGH, which physiologically antagonizes insulin action, likely acted as a metabolic stressor, exacerbating the underlying predisposition linked both to the SGA status and the *IGF1R* defect. This underscores the importance of close metabolic surveillance in SGA children, especially when a genetic receptor resistance coexists. However, the role of rhGH therapy as a potential trigger for glucose metabolism alterations in SGA individuals carrying *IGF1R* mutations requires confirmation through further studies on larger cohorts. Consequently, our findings cannot be generalized to all patients with *IGF1R* mutations undergoing rhGH therapy.

## 4. Conclusions

In conclusion, this case reports a novel *IGF1R* mutation and highlights the critical role of genetic testing in the evaluation of SGA patients. Such a diagnosis provides a clear explanation for a poor response to rhGH therapy and has significant implications for clinical management. Specifically, the decision to initiate or continue rhGH treatment should consider that growth outcomes in individuals with *IGF1R* defects are significantly blunted compared to those in SGA children with SS without this genetic defect. Furthermore, clinicians should be aware of the ‘*dual burden*’ of the IGF1R mutation and the SGA status, as these two potentially concurrent factors together increase the susceptibility to alterations in glucose metabolism; however, this possibility warrants a case-by-case evaluation and should be further validated through studies on larger cohorts.

## Figures and Tables

**Table 1:** Auxological and biochemical data of our case.

Age (Years)	Height (cm)	Height SDS	Weight (kg)	Weight SDS	BMI (kg/m^2^)	BMI SDS	Bone Age (Years)	Growth Rate cm/y (SDS)	Tanner Stage	IGF-1 SDS
9.47	112.10	−3.99	18.20	−3.64	14.48	−1.65	8	3.74 (−2.11)	P1G1	+1.7
Start GH therapy (0.035 mg/kg/day)										
10.59	119.10	−3.71	21.50	−3.11	15.16	−1.46	9	6.21 (+1.59)	P1G1	+3.3
11.59	124.40	−3.56	23	−3.24	14.86	−1.89	9.90	5.29 (+0.38)	P1G1	
12.59	128.80	−3.46	25.30	−3.27	15.25	−1.93	10.90	4.42 (−1.12)	P1G1	+1.3

## Data Availability

The original contributions presented in this study are included in the article. Further inquiries can be directed to the corresponding author.

## Supplementary Materials

The following supporting information can be downloaded at: https://www.mdpi.com/article/10.3390/children13040458/s1, Table S1: Overview of growth rate during GH treatment in cases of SGA with IGF1R gene alterations.
