# Supplementary material for: Severe Short Stature and rhGH Resistance in a Child Born SGA: The Role of a Novel IGF1R Mutation, Case Report and Narrative Review
Source: Children (Basel). 2026 Mar 27;13(4):458. doi: 10.3390/children13040458 (PMC13115005; doi:10.3390/children13040458)
Supplement: Supplementary file 1 [file children-13-00458-s001.zip › children-4170532-supplementary.pdf]

Table S1. Overview of growth rate during GH treatment in cases of SGA with IGF1R gene alterations.

References	[16]		[23]	[24]	[25]		[26]	[27]	[28]	[29]	Our case
Patient	Girl	Boy	Girl	Boy	Boy	Boy	Girl	Girl	Girl	Boy	Boy
Genetic variant	Deletion of 15q26.2	Deletion of 15q26.3	Deletion of 15q26.2	IGF1R variant Ala760Glyfs*21	IGF1R variant p.Cys1248Tyr	IGF1R variant p.Cys1248Tyr	Deletion of 15q26.2q26	IGF1R variant p.M1247T	Deletion of 15q26.3	IGF1R variant p.Tyr387X	IGF1R variant p.Tyr1166Ter
Age gestational (weeks + days)	40	40	39	At term	39 + 1/7	39 + 6/7	39 + 1/7	38	38	At term	39
Birth length (SDS)	−2.21	−2.74	−1.4	-	−2.61	−1.56	-	-	-	−3.08	−2.81
Birth weight (SDS)	−1.28	−1.91	−3.0	−2.5	−3.18	−2.08	−4.18	−1.74	−2.5	−2.03	−1.66
Age (years) at GH treatment initiation	4	7	5.3	6	5	6	6	2	1	8	9
Height (SDS) at GH treatment initiation	−3.42	−3.57	−3.5	−3.4	−3.73	−3.06	−4.26	−3.85	−3.3	-	−3.99
Serum IGF-1 level (SDS) at GH treatment initiation	+2.78	+1.25	+2.5	+0.95	+0.88	+1.53	−0.66	+1.4	+0.9	+2.1 §	+1.7
GH therapy dose (mg/kg/day)	1 ^	1 ^	1 ^	0.035	0.027	0.027	0.032	0.042	0.04	0.037	0.035
Height (SDS) after 2 years of therapy	−2.40 °	−2.74 °	−1.6 **	−2.8	−2.92	−2.48	−3.12	HV changed from less than 3 SD to less than 2 SD at 8 years of age	HV was 3.84 cm/year in 9-month of therapy	No improvement in HV	−3.46 SDS. Total height recovery of only 0.5 SDS °°°
Serum IGF1 levels (SDS) after 1 year of therapy	+1.51	+2.28	+3.5	+5 °°	+2.56	+3.09	+4.63	-	+0.64 ***	-	+1.3 °°°
GH deficiency	no	no	no	no	no	no	yes	no	no	no	no
Altered Glucose Metabolism	no	no	no	no	IGT	IGT	no	no	no	IGT	IFG and IGT

^ mg/m^2/day; ° after one year of therapy; °° after two years of therapy; °°° after three years of therapy; ** Adult height; *** after nine months of therapy; § At the age of 6 years; HV: height velocity; IFG: impaired fasting glycemia; IGT: impaired glucose tolerance; SDS: standard deviation scores (SDS), GH: growth hormone.
